# Supplementary material for: Prediction of m6A and m5C at single-molecule resolution reveals a transcriptome-wide co-occurrence of RNA modifications
Source: Nat Commun. 2024 May 9;15:3899. doi: 10.1038/s41467-024-47953-7 (PMC11082244; doi:10.1038/s41467-024-47953-7)
Supplement: Supplementary file 3 — Description of Additional Supplementary Files [file 41467_2024_47953_MOESM3_ESM.pdf]

Description of Additional Supplementary Files:

Supplementary Data 1: Summary of the samples used. We indicate the number of reads that were classified as pass or fail by Guppy and the total number of reads mapped to the human (or mouse) transcriptome considering primary alignments (see Methods), both in total counts and as a proportion of the initial number of reads.

Supplementary Data 2: Significant sites detected by CHEUI-solo in each condition. For each condition, we give the number of transcripts that were tested, the number of transcriptomic sites tested (which had at least 20 reads in that condition), and the number of significant sites identified by CHEUI-solo Model 2 using a probability cutoff of P>0.9999, selected by controlling for the FPR with a permutation test, as described in the Methods section.

Supplementary Data 3: Significant sites from CHEUI-solo predictions in HEK293 WT. Column 1: Chromosome; column 2: genomic start position (half-open) of the modified site; column 3: genomic end position of the modified site; column 4: transcript unique ID; column 5: score (not used); column 6: genomic strand; column 7: Transcript position (half-open) of the modified nucleotide; column 8: site motif; column 9: site coverage (number of reads); column 10: site stoichiometry; column 11: modified site probability; column 12: Transcript type; column 13: Gene name; column 14: Gene unique ID; column 15: transcript length (nts); column 16: transcript CDS length (nts); column 17: transcript 5'UTR length (nts); column 18: 3'UTR length (nts); column 19: CDS start in transcript coordinates; column 20: CDS end in transcript coordinates; column 21: site position relative to the transcript segments (see Methods). Ranges from 0 to 3. From 0-1 corresponds to start and end of 5'UTR; 1-2 corresponds to start and end of CDS, 2-3 corresponds to start and end of 3'UTR; column 22: abs_cds_start, absolute distance in nucleotides of the site from the annotated start of the CDS; column 23: abs_cds_end absolute distance in nucleotides of the site from the annotated end of the CDS; column 23: distance from the site to the closest junction upstream; column 24: distance from the site to the closest junction downstream; column 25: Sample name column 26: modification (m6A or m5C).

Supplementary Data 4: Significant sites from CHEUI-solo m6A predictions in HEK293 METTL3-KO sample. Column 1: Chromosome; column 2: genomic start position (half-open) of the modified site; column 3: genomic end position of the modified site; column 4: transcript unique ID; column 5: score (not used); column 6: genomic strand; column 7: Transcript position (half-open) of the modified nucleotide; column 8: site motif; column 9: site coverage (number of reads); column 10: site stoichiometry; column 11: modified site probability; column 12: Transcript type; column 13: Gene name; column 14: Gene unique ID; column 15: transcript length (nts); column 16: transcript CDS length (nts); column 17: transcript 5'UTR length (nts); column 18: 3'UTR length (nts); column 19: CDS start in transcript coordinates; column 20: CDS end in transcript coordinates; column 21: site position relative to the transcript segments (see Methods). Ranges from 0 to 3. From 0-1 corresponds to start and end of 5'UTR; 1-2 corresponds to start and end of CDS, 2-3 corresponds to start and end of 3'UTR; column 22: abs_cds_start, absolute distance in nucleotides of the site from the annotated start of the CDS; column 23: abs_cds_end absolute distance in nucleotides of the site from the

annotated end of the CDS; column 23: distance from the site to the closest junction upstream; column 24: distance from the site to the closest junction downstream; column 25: Sample name; column 26: mnodification (m6A).

Supplementary Data 5: Significant differential m6A sites predicted by CHEUI-diff between WT and METTL3-KO in HEK293. Column 1: transcript site unique ID, column 2: coverage of site in condition 1 (WT) , column 3: coverage of site in condition 2 (METTL3-KO), column 4: stoichiometry in condition 1, column 5: stoichiometry in condition 2, column 6: the absolute difference between the two conditions stoichiometries, column 7: The test statistic under the large-sample approximation that the rank sum statistic is normally distributed, column 8: p-value from the two-tailed Mann-Whitney U-test, column 9: FDR-adjusted p-value (also called q-value) of 0.05.

Supplementary Data 6: Significant sites from CHEUI-solo predictions in HeLa WT sample. Column 1: Chromosome; column 2: genomic start position (half-open) of the modified site; column 3: genomic end position of the modified site; column 4: transcript unique ID; column 5: score (not used); column 6: genomic strand; column 7: Transcript position (half-open) of the modified nucleotide; column 8: site motif; column 9: site coverage (number of reads); column 10: site stoichiometry; column 11: modified site probability; column 12: Transcript type; column 13: Gene name; column 14: Gene unique ID; column 15: transcript length (nts); column 16: transcript CDS length (nts); column 17: transcript 5'UTR length (nts); column 18: 3'UTR length (nts); column 19: CDS start in transcript coordinates; column 20: CDS end in transcript coordinates; column 21: site position relative to the transcript segments (see Methods). Ranges from 0 to 3. From 0-1 corresponds to start and end of 5'UTR; 1-2 corresponds to start and end of CDS, 2-3 corresponds to start and end of 3'UTR; column 22: abs_cds_start, absolute distance in nucleotides of the site from the annotated start of the CDS; column 23: abs_cds_end absolute distance in nucleotides of the site from the annotated end of the CDS; column 23: distance from the site to the closest junction upstream; column 24: distance from the site to the closest junction downstream; column 25: Sample name; column 26: modification (m6A or m5C).

Supplementary Data 7: Significant sites from CHEUI-solo m5C predictions in HeLa NSUN2-KO sample. Column 1: Chromosome; column 2: genomic start position (half-open) of the modified site; column 3: genomic end position of the modified site; column 4: transcript unique ID; column 5: score (not used); column 6: genomic strand; column 7: Transcript position (half-open) of the modified nucleotide; column 8: site motif; column 9: site coverage (number of reads); column 10: site stoichiometry; column 11: modified site probability; column 12: Transcript type; column 13: Gene name; column 14: Gene unique ID; column 15: transcript length (nts); column 16: transcript CDS length (nts); column 17: transcript 5'UTR length (nts); column 18: 3'UTR length (nts); column 19: CDS start in transcript coordinates; column 20: CDS end in transcript coordinates; column 21: site position relative to the transcript segments (see Methods). Ranges from 0 to 3. From 0-1 corresponds to start and end of 5'UTR; 1-2 corresponds to start and end of CDS, 2-3 corresponds to start and end of 3'UTR; column 22: abs_cds_start, absolute distance in nucleotides of the site from the annotated start of the CDS; column 23: abs_cds_end absolute distance in nucleotides of the site from the annotated end of the CDS; column 23: distance from the site to the closest junction upstream;

column 24: distance from the site to the closest junction downstream; column 25: Sample name; column 26: model (m5C).

Supplementary Data 8: Significant sites CHEUI-diff predictions differential m5C between WT and NSUN2-KO in HeLa cells. Column 1: transcript site unique ID, column 2: coverage of site in condition 1 (WT), column 3: coverage of site in condition 2 (NSUN2-KO), column 4: stoichiometry in condition 1, column 5: stoichiometry in condition 2, column 6: the absolute difference between the two conditions stoichiometries, column 7: The test statistic under the large-sample approximation that the rank sum statistic is normally distributed, column 8: p-value from the two-tailed Mann-Whitney U-test, column 9: FDR-adjusted p-value (also called q-value) of 0.05.

Supplementary Data 9: Comparison of bsRNA-seq and CHEUI with IVTs. (a) IVT sequences used for the comparison of bsRNA-seq and CHEUI in the identification of m5C. (b) Coverage of the C sites by nanopore and bsRNA-seq. (c) Recall and False Positive Rate (FPR) accomplished by CHEUI

Supplementary Data 10: Significant sites CHEUI-solo predictions of m6A and m5C in three developmental time points from mouse prefrontal cortex. Column 1: Chromosome; column 2: genomic start position (half-open) of the modified site; column 3: genomic end position of the modified site; column 4: transcript unique ID; column 5: score (not used); column 6: genomic strand; column 7: Transcript position (half-open) of the modified nucleotide; column 8: site motif; column 9: site coverage (number of reads); column 10: site stoichiometry; column 11: modified site probability; column 12: Transcript type; column 13: Gene name; column 14: Gene unique ID; column 15: transcript length (nts); column 16: transcript CDS length (nts); column 17: transcript 5’UTR length (nts); column 18: 3’UTR length (nts); column 19: CDS start in transcript coordinates; column 20: CDS end in transcript coordinates; column 21: site position relative to the transcript segments (see Methods). Ranges from 0 to 3. From 0-1 corresponds to start and end of 5’UTR; 1-2 corresponds to start and end of CDS, 2-3 corresponds to start and end of 3’UTR; column 22: abs_cds_start, absolute distance in nucleotides of the site from the annotated start of the CDS; column 23: abs_cds_end absolute distance in nucleotides of the site from the annotated end of the CDS; column 23: distance from the site to the closest junction upstream; column 24: distance from the site to the closest junction downstream; column 25: modification (m6A or m5C) ; column 26: Sample name.

Supplementary Data 11: Summary of number of different 9-mers and signals used for training (IVT train 1), validation (IVT validation 1), and testing (IVT test 1, IVT test 2) of the m6A and m5C models.
