# Supplementary material for: Prediction of m6A and m5C at single-molecule resolution reveals a transcriptome-wide co-occurrence of RNA modifications
Source: Nat Commun. 2024 May 9;15:3899. doi: 10.1038/s41467-024-47953-7 (PMC11082244; doi:10.1038/s41467-024-47953-7)
Supplement: Supplementary file 15 — Reporting Summary [file 41467_2024_47953_MOESM15_ESM.pdf]

nature portfolio

Corresponding author(s): Nikolay E. Shirokikh, Eduardo Eyras

Last updated by author(s): Mar 8, 2024

Reporting Summary

Nature Portfolio wishes to improve the reproducibility of the work that we publish. This form provides structure for consistency and transparency in reporting. For further information on Nature Portfolio policies, see our Editorial Policies and the Editorial Policy Checklist.

Statistics

For all statistical analyses, confirm that the following items are present in the figure legend, table legend, main text, or Methods section.

n/a	Confirmed	
☐	☒	The exact sample size (*n*) for each experimental group/condition, given as a discrete number and unit of measurement
☐	☒	A statement on whether measurements were taken from distinct samples or whether the same sample was measured repeatedly
☐	☒	The statistical test(s) used AND whether they are one- or two-sided<br>*Only common tests should be described solely by name; describe more complex techniques in the Methods section.*
☒	☐	A description of all covariates tested
☐	☒	A description of any assumptions or corrections, such as tests of normality and adjustment for multiple comparisons
☐	☒	A full description of the statistical parameters including central tendency (e.g. means) or other basic estimates (e.g. regression coefficient) AND variation (e.g. standard deviation) or associated estimates of uncertainty (e.g. confidence intervals)
☐	☒	For null hypothesis testing, the test statistic (e.g. *F*, *t*, *r*) with confidence intervals, effect sizes, degrees of freedom and *P* value noted<br>*Give P values as exact values whenever suitable.*
☒	☐	For Bayesian analysis, information on the choice of priors and Markov chain Monte Carlo settings
☒	☐	For hierarchical and complex designs, identification of the appropriate level for tests and full reporting of outcomes
☐	☒	Estimates of effect sizes (e.g. Cohen's *d*, Pearson's *r*), indicating how they were calculated

Our web collection on statistics for biologists contains articles on many of the points above.

Software and code

Policy information about availability of computer code

Data collection	wget (freely available from the GNU project under the GPL-v3 license), SRA toolkit (freely available from https://github.com/ncbi/sra-tools under a public domain license)
Data analysis	CHEUI https://github.com/comprna/CHEUI<br>We have provided a copy of the CHEUI code and documentation at DOI: 10.5281/zenodo.7021308<br>R2Dtool (v1) : https://github.com/comprna/R2Dtool<br>Nanocompore (v1.0.0rc3-2): https://github.com/tleonardi/nanocompore<br>Xpore (v0.5.4): https://github.com/GoekeLab/xpore<br>Epinano (v 0.1-2020-04-04): https://github.com/novoalab/EpiNano<br>Tombo (v1.5): https://github.com/nanoporetech/tombo<br>NanoRMS (v1): https://github.com/novoalab/nanoRMS<br>Keras (v1.1.2): https://github.com/keras-team/keras<br>Tensorflow (v2.4.1): https://github.com/tensorflow<br>Minimap2 (v2.1.0): https://github.com/lh3/minimap2<br>Nanopolish (v0.13.2): https://github.com/jts/nanopolish<br>RNAfold (v2.4.18): https://www.tbi.univie.ac.at/RNA/

For manuscripts utilizing custom algorithms or software that are central to the research but not yet described in published literature, software must be made available to editors and reviewers. We strongly encourage code deposition in a community repository (e.g. GitHub). See the Nature Portfolio guidelines for submitting code & software for further information.

Data

Policy information about availability of data

All manuscripts must include a data availability statement. This statement should provide the following information, where applicable:

- Accession codes, unique identifiers, or web links for publicly available datasets
- A description of any restrictions on data availability
- For clinical datasets or third party data, please ensure that the statement adheres to our policy

All datasets used in this publication are publicly available. The synthetic sequence templates from Liu et al. 2009 were obtained from the NCBI Gene Expression Omnibus (GEO) database under the accession number GSE124309. The nanopore read signals for the in-vitro transcribed (IVT) RNAs obtained from these synthetic sequence templates with m6A, m5C, or no modifications, were obtained from NCBI Sequence Read Archive (SRA) under accessions PRJNA511582 and PRJNA563591. Nanopore data for the synthetic transcripts from Jenjaroepun et al. 2021 was obtained from The Sequence Read Archive (SRA) accession PRJNA497103. Nanopore data for HEK293 WT and METTL3-KO samples from Pratanwanich et al. 2021 was obtained from the European Nucleotide Archive (ENA) under accession PRJEB40872. Data from the m6ACE-seq experiments from Koh et al. 2019 was obtained from the NCBI Gene Expression Omnibus (GEO) under accession number GSE124509. Nanopore data for HeLa WT and HeLa NSUN2 KO and for the embryonic mouse brain tissues produced in this work have been deposited at NCBI GEO under accession GSE211762. Nanopore sequencing and bisulfite RNA sequencing data for the IVT RNAs is available at NCBI GEO under accession GSE253150. All source data files for the main and supplementary figures for this publication are publicly available in figshare [10.6084/m9.figshare.25424857].

Research involving human participants, their data, or biological material

Policy information about studies with human participants or human data. See also policy information about sex, gender (identity/presentation), and sexual orientation and race, ethnicity and racism.

Reporting on sex and gender	N/A
Reporting on race, ethnicity, or other socially relevant groupings	N/A
Population characteristics	N/A
Recruitment	N/A
Ethics oversight	N/A

Note that full information on the approval of the study protocol must also be provided in the manuscript.

Field-specific reporting

Please select the one below that is the best fit for your research. If you are not sure, read the appropriate sections before making your selection.

[x] Life sciences [ ] Behavioural & social sciences [ ] Ecological, evolutionary & environmental sciences

For a reference copy of the document with all sections, see nature.com/documents/nr-reporting-summary-flat.pdf

Life sciences study design

All studies must disclose on these points even when the disclosure is negative.

Sample size	No sample size was calculated. We used three biological replicates per condition with cell lines, and two or more for mouse embryonic tissues, which allowed us to estimate the experimental and biological variability in each condition.
Data exclusions	Modifications occurring in ribosomal RNA (rRNA) were excluded from this study, as the ribosomal RNA is known to be hypermodified with a large variety of modifications that, as we show in the manuscript, can affect the detection of m6A and m5C. As only reads for one rRNA (18S) in mouse were observed, this exclusion does not impact the general results of our analyses.
Replication	We used two or three replicates for each of the experiments and show in the manuscript that our analyses are consistent across replicates. This is shown for the cell lines and the mouse tissues.
Randomization	Randomization was performed at the time of splitting the available in vitro transcribed datasets into training, validation, and testing. This was performed only once. Additional randomization of reads and positions was performed once per dataset to estimate the false discovery rate. For the selection of mouse samples, randomization was not performed. Randomization was also performed when selecting mouse embryos for RNA sequencing: male and female embryos were randomly selected for each condition. Conditions were known and comparisons were performed between conditions, so no further randomization was done. No other covariates were used for the analyses of IVT, cell line, or mouse tissue experiments.
Blinding	Blinding was not performed in this study, since the sequencing was performed of samples in known conditions, such as a wild type cell or a knckout. Knowing the condition was essential for the study.

Reporting for specific materials, systems and methods

We require information from authors about some types of materials, experimental systems and methods used in many studies. Here, indicate whether each material, system or method listed is relevant to your study. If you are not sure if a list item applies to your research, read the appropriate section before selecting a response.

Materials & experimental systems

n/a	Involved in the study	
☐	☒	Antibodies
☐	☒	Eukaryotic cell lines
☒	☐	Palaeontology and archaeology
☐	☒	Animals and other organisms
☒	☐	Clinical data
☒	☐	Dual use research of concern
☒	☐	Plants

Methods

n/a	Involved in the study	
☒	☐	ChIP-seq
☐	☒	Flow cytometry
☒	☐	MRI-based neuroimaging

Antibodies

Antibodies used	anti-NSUN2 (1:1000; Proteintech, cat. no. 20854-1-AP), anti-ACTB (1:1000; SantaCruz, cat. no. sc-47778 AF790), anti-rabbit-IR-Dye680 secondary antibody (1:10,000; LI-COR, cat. no. 925-68071)
Validation	anti-NSUN2 antibody validation available from https://www.ptglab.com/products/NSUN2-Antibody-20854-1-AP.htm<br>anti-ACTB antibody validation available from https://www.scbt.com/p/beta-actin-antibody-c4

Eukaryotic cell lines

Policy information about cell lines and Sex and Gender in Research

Cell line source(s)	HeLa cells (human cervical cancer) were obtained from ATCC (cat. no CCL-2)
Authentication	HeLa cells were confirmed via short tandem repeat (STR) profiling with CellBank Australia.
Mycoplasma contamination	HeLa cell cultures were negative for mycoplasma contamination in a test prior to their processing for gene editing. Additionally, cells were grown in medium supplemented an antibiotic-antimycotic solution (Sigma).
Commonly misidentified lines<br>(See ICLAC register)	No commonly misidentified cell lines were used in this study.

Animals and other research organisms

Policy information about studies involving animals; ARRIVE guidelines recommended for reporting animal research, and Sex and Gender in Research

Laboratory animals	species: mouse, strain: C57BL/6J, age: embryonic (E12, E15, E18). Mice were collectively housed in standard Makrolon cages, in a temperature- and humidity-controlled room under a 12h light/dark cycle (lights on at 07:00), with ad libitum access to food and water.
Wild animals	No wild animals were used in this study.
Reporting on sex	All mouse cortex samples were obtained sampling randomly male and female embryos. Samples from the same embryonic stage were pooled. Sex of the embryonic samples was not reported.
Field-collected samples	No field collected samples were used in this study.
Ethics oversight	All procedures were conducted in accordance with the Australian National University Animal Experimentation Ethics Committee (protocol number A2019/46)

Note that full information on the approval of the study protocol must also be provided in the manuscript.

Plants

Seed stocks	*Report on the source of all seed stocks or other plant material used. If applicable, state the seed stock centre and catalogue number. If plant specimens were collected from the field, describe the collection location, date and sampling procedures.*
Novel plant genotypes	*Describe the methods by which all novel plant genotypes were produced. This includes those generated by transgenic approaches, gene editing, chemical/radiation-based mutagenesis and hybridization. For transgenic lines, describe the transformation method, the number of independent lines analyzed and the generation upon which experiments were performed. For gene-edited lines, describe the editor used, the endogenous sequence targeted for editing, the targeting guide RNA sequence (if applicable) and how the editor was applied.*
Authentication	*Describe any authentication procedures for each seed stock used or novel genotype generated. Describe any experiments used to assess the effect of a mutation and, where applicable, how potential secondary effects (e.g. second site T-DNA insertions, mosiacism, off-target gene editing) were examined.*

Flow Cytometry

Plots

Confirm that:

- ☐ The axis labels state the marker and fluorochrome used (e.g. CD4-FITC).
- ☐ The axis scales are clearly visible. Include numbers along axes only for bottom left plot of group (a 'group' is an analysis of identical markers).
- ☐ All plots are contour plots with outliers or pseudocolor plots.
- ☐ A numerical value for number of cells or percentage (with statistics) is provided.

Methodology

Sample preparation	*Describe the sample preparation, detailing the biological source of the cells and any tissue processing steps used.*
Instrument	*Identify the instrument used for data collection, specifying make and model number.*
Software	*Describe the software used to collect and analyze the flow cytometry data. For custom code that has been deposited into a community repository, provide accession details.*
Cell population abundance	*Describe the abundance of the relevant cell populations within post-sort fractions, providing details on the purity of the samples and how it was determined.*
Gating strategy	*Describe the gating strategy used for all relevant experiments, specifying the preliminary FSC/SSC gates of the starting cell population, indicating where boundaries between "positive" and "negative" staining cell populations are defined.*

☐ Tick this box to confirm that a figure exemplifying the gating strategy is provided in the Supplementary Information.
